# Sleep architecture in Alzheimer’s disease continuum: The deep sleep question

**DOI:** 10.1515/biol-2025-1077

**Published:** 2025-03-25

**Authors:** Ioannis Foukarakis, Stefanos N. Sampatakakis, Eirini Mamalaki, Andreas Kyrozis, Eva Ntanasi, Angeliki Tsapanou, Mary Yannakoulia, Konstantinos Rouskas, Nikolaos Scarmeas

**Affiliations:** 1st Department of Neurology, Aiginition Hospital, National and Kapodistrian University of Athens Medical School, 11528, Athens, Greece; The Gertrude H. Sergievsky Center, Taub Institute for Research in Alzheimer’s Disease and the Aging Brain, Department of Neurology, Columbia University, New York, NY, 10032, United States of America; Department of Nutrition and Dietetics, Harokopio University, 17671 Athens, Greece; Institute of Applied Biosciences, Centre for Research & Technology Hellas, 54124 Thessaloniki, Greece; Department of Speech and Language Therapy, School of Health Rehabilitation Sciences, University of Patras, Patras, 26504, Greece

**Keywords:** mild cognitive impairment, CSF biomarkers, amyloid-beta 42, sleep stages, WatchPAT

## Abstract

As sleep appears to be closely related to cognitive status, we aimed to explore the association between the percentage of deep sleep, cognitive state, and the cerebrospinal fluid (CSF) biomarker amyloid-beta 42 in non-demented individuals. In this cross-sectional study, 90 non-demented participants from the Aiginition Longitudinal Biomarker Investigation of Neurodegeneration cohort underwent a one-night WatchPAT sleep evaluation. Participants were categorized by cognitive status (patients with mild cognitive impairment [MCI] or cognitively normal [CN] individuals) and CSF Aβ42 status (Aβ42 ≤ 1,030 pg/mL [A+] or Ab42 > 1,030 pg/mL [A−]). After controlling for age, sex, and years of education, a significant inverse association was found between the percentage of deep sleep and the odds of being classified as MCI compared to CN (OR = 0.86, 95% CI [0.76–0.97], *p* = 0.012). However, a non-significant trend for an inverse association between the percentage of deep sleep and the odds of being classified as A+ was observed (OR = 0.92, 95% CI [0.84–1.01], *p* = 0.092). This study demonstrates a significant link between deep sleep and MCI. Although more longitudinal studies are needed, deep sleep could potentially serve as a novel biomarker of cognitive decline and an intervention target for dementia prevention.

## Graphical abstract

**Figure:**
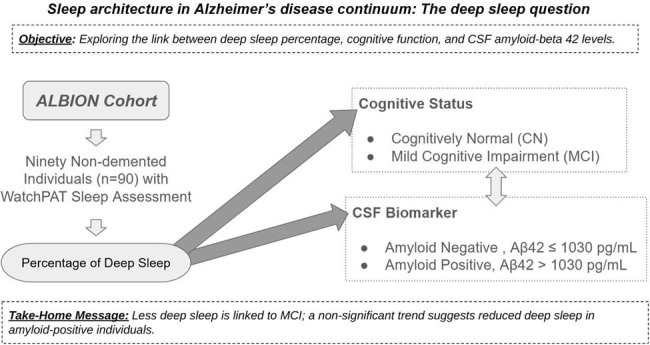

## 1 Introduction

In 2023, dementia was the seventh leading cause of death and one of the major causes of disability among older adults globally, while Alzheimer’s disease (AD) is the most common form of dementia, representing 60–70% of all cases [1]. Between dementia and normal cognitive aging there is an intermediate stage, namely mild cognitive impairment (MCI) [2]. Although MCI can result from a variety of diseases, AD accounts for many cases, thus MCI constitutes a well-recognized part of AD clinical continuum [2]. At the same time, AD biomarkers, and especially amyloid-beta (Aβ42), which can precede clinical symptoms by years [3], are considered a hallmark in AD pathophysiological continuum [4]. Consequently, research interest has shifted to these preclinical and prodromal stages of the disease and the possible associations with lifestyle factors.

Regarding lifestyle factors, apart from diet [5] and physical activity [6], cognitive decline is also closely associated with sleep [7]. Notably, the prevalence of sleep disturbances in patients with pathological cognitive decline is significantly higher compared to cognitively normal (CN) individuals [8], while numerous cross-sectional and prospective studies have demonstrated that poor sleep quality is linked to cognitive impairment [7,9,10]. However, existing evidence suggests a bidirectional relationship between sleep and AD pathology [11,12]. Beyond overall sleep quality and self-reported sleep disturbances, a particularly intriguing area of research is deep sleep, also known as slow wave sleep (SWS) or N3 (non-rapid eye movement, NREM) sleep, and its impact on cognition. Its crucial role in the consolidation of declarative memories has been firmly established in healthy older adults [13,14], although the exact underlying physiology of this procedure remains debated [14]. In CN older adults, SWS has been positively associated with cognitive functions, especially episodic memory performance [15], whereas several studies suggest that enhancing deep sleep activity or duration may lead to performance improvements in various cognitive domains [16].

However, data regarding the association between deep sleep and MCI have provided conflicting results. Some cross-sectional studies have provided evidence of such a relationship [17,18,19,20], while other studies have failed to detect any significant correlation [21,22,23,24,25,26]. Two meta-analyses [27,28] which have compared the sleep macroarchitecture in CN and MCI subjects, concluded that there were no significant differences between the two groups regarding deep sleep. As far as the association between amyloid-beta burden and deep sleep in non-demented individuals is concerned, there are a few studies dealing with this topic. In patients with amnestic MCI (aMCI) a positive correlation between disrupted deep sleep and plasma Aβ42 levels has been documented [29], while in cognitively healthy individuals a link between diminished deep sleep duration and cerebrospinal fluid (CSF) Aβ42 was found [30].

Nonetheless, the above-mentioned studies are not without limitations. First, the majority of studies concerning cognitive status included a low number of non-demented participants (less than 50) [17,18,19,23,24,26], limiting their power. Additionally, in many studies, participants were on average over 70 years old [17,18,19,20,26], indicating the need for more comprehensive data on younger non-demented individuals within the AD continuum and only two of them have presented data regarding the participants’ amyloid-beta status [25,26]. Studies regarding the relationship between amyloid-beta and deep sleep included a small number of participants (less than 50) as well, while Aβ42 evaluation was either performed through plasma [29], where there is no consensus for the cut-offs criteria [31], or did not include individuals with pathological Aβ42 values [30].

Within this theoretical framework, our aim in undertaking this specific cross-sectional study was to enhance current understanding of sleep macroarchitecture in non-demented people, placing emphasis on deep sleep percentage. Specifically, our goal was to investigate differences in the percentage of deep sleep, as measured by a WatchPAT device, among CN individuals and patients with MCI and to explore the association between the fraction of deep sleep and amyloid-beta burden.

## 2 Materials and methods

### 2.1 Participants and study design

Data for the analysis were drawn from Aiginition Longitudinal Biomarker Investigation of Neurodegeneration (ALBION) study, a longitudinal ongoing study in the Cognitive Disorders Clinic of Aiginition Hospital of the National and Kapodistrian University of Athens [32]. Our sample consisted of individuals aged ≥40 years, referred by other specialists or self-referred to the cognitive disorders’ outpatient clinic of Aiginition, Athens, Greece. Patients with neurological and psychiatric diseases, including dementia, Parkinson’s disease, multiple sclerosis, Huntington’s disease, severe traumatic brain injury, and normal pressure hydrocephalus, as well as those with active alcohol or drug abuse, major psychiatric conditions such as major depressive disorder, schizophrenia, and bipolar disorder, or other medical conditions associated with cognitive impairment, were excluded from ALBION. All subjects underwent a standardized physical and neurological examination, comprehensive neuropsychological assessment, a lumbar puncture, blood sampling, and one-night sleep activity assessment by WatchPAT as part of their baseline evaluation. All participants provided written informed consent at the time of enrollment. More details regarding ALBION study protocol can be found elsewhere [32].

Initially, a total of 116 participants in the ALBION cohort underwent sleep evaluation using a WatchPAT device. Five participants provided incomplete data because they did not adhere to the instructions for proper WatchPAT measurement, which impacted the calculated total sleep time (TST) (e.g., WatchPAT activation failure, incomplete measurements, or activation occurring extremely early in the morning). Furthermore, five participants diagnosed with obstructive sleep apnea syndrome by a polysomnography study and subsequently treated with continuous positive airway pressure device were excluded from this study. Also, considering the potential influence of benzodiazepines on sleep macro-architecture [33], particularly on deep sleep, we excluded 16 participants who were systematically treated with benzodiazepines.

**Figure 1:**
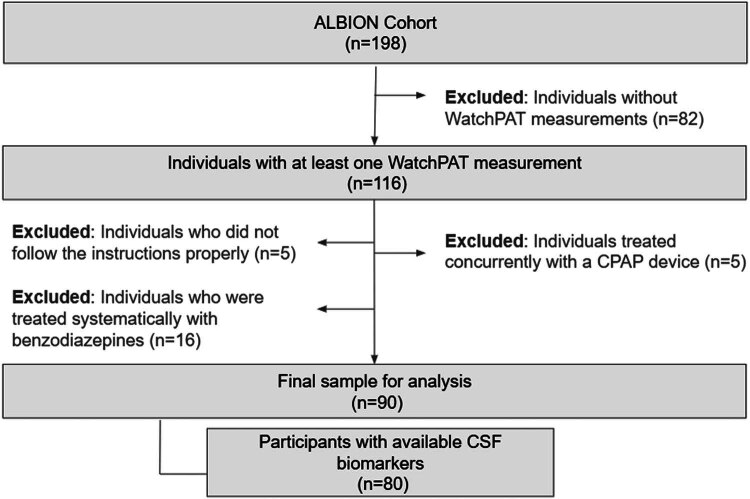
Flowchart.


In total, our study consisted of 90 participants, with CSF analysis data available for 80, as depicted in our flowchart (Figure 1).

**Informed consent:** Informed consent has been obtained from all individuals included in this study.
**Ethical approval:** The research related to human use has been complied with all the relevant national regulations, institutional policies and in accordance with the tenets of the Helsinki Declaration, and has been approved by the Institutional Review Board and Ethics Committee of the Aiginition University Hospital, National and Kapodistrian University of Athens, Greece (Protocol code: 255, A∆A: ΨΘ6K46Ψ8N2-8HΩ, date of approval: 10 May 2021).

### 2.2 Clinical diagnosis, neurological, and neuropsychological assessment

All participants have successfully completed a thorough standardized neuropsychological assessment conducted by trained clinical neuropsychologists who administered a battery of neuropsychological tests assessing five cognitive domains: attention-speed, executive functioning, memory, language, and visuospatial perception. Global cognition was assessed using Mini Mental State Examination (MMSE) [34] and the Revised Addenbrooke’s Cognitive Examination (ACE-R) [35]. Further information regarding the neuropsychological evaluation in ALBION study can be found elsewhere [32]. Subsequently, a neurological examination was performed by a specialist neurologist who recorded detailed information regarding demographics, medical history, medication, and family history.

A diagnosis of MCI and MCI subtypes (aMCI and non-amnestic [naMCI]) was assigned using standard criteria [36] when the participant had cognitive complaints and a measurable deficit in cognition with a standard deviation (SD) below 1.5 in at least one domain, in the absence of dementia or impairment in everyday functioning.

We defined cognitive status (either CN or MCI) of each participant based on the clinical status corresponding to the visit when they successfully completed the WatchPAT evaluation.

### 2.3 CSF collection and analysis

Eighty (*n* = 80) participants underwent a lumbar puncture during their first visit, according to ALBION’s study protocol [32], and had available CSF data by the time of our analysis. The lumbar puncture procedure, as well as the collection, processing, and storage of CSF, followed international guidelines [37]. CSF was primarily processed for Aβ42 biomarker, which is indicative of AD. Analysis of CSF neurodegenerative biomarkers was performed using the Roche Diagnostics Elecsys© Platform (Elecsys® β-Amyloid (1-42) CSF II). The provided reference ranges for a positive result were as follows: Aβ42 ≤ 1,030 pg/mL based on the established cut-off explicitly provided by the assay manufacturer [38]. Participants were classified as amyloid positive (A+) if the CSF Aβ42 value was less than 1,030 pg/mL; otherwise, they were classified as amyloid negative (A−).

### 2.4 Sleep measures

The WatchPAT device is an objective, battery-powered, wrist-worn ambulatory sleep recorder equipped with sensors that detect peripheral arterial tone (PAT) signal, pulse rate, actigraphy, and pulse oximetry. The PAT signal measures pulsatile volume changes at the fingertip, reflecting variations in sympathetic tone. The recorded signals from the WatchPAT device are automatically analyzed using advanced software, which categorizes sleep into wake, light sleep, deep sleep, and rapid eye movement (REM) sleep [39,40]. This classification is based on evaluations of body movements and their occurrence, as well as spectral components of the PAT signal. The WatchPAT algorithm stratifies sleep into light, deep, and REM stages, with light sleep corresponding to sleep stages N1 and N2, and deep sleep corresponding to sleep stage N3. Further details on the algorithm can be found elsewhere [39,40,41]. A multicenter validation study comparing the WatchPAT device to polysomnography (PSG) demonstrated an overall agreement of 88.6 ± 5.9% for detecting light/deep sleep and 88.7 ± 5.5% for detecting REM sleep across a varied population [42]. Cohen *κ* coefficients were calculated to adjust for random agreement and suggested moderate agreement between polysomnography and WatchPAT recordings [43].

In the current study, WatchPAT300 device was used to assess participants’ nighttime sleep macroarchitecture, focusing on TST, sleep latency, number of awakenings, and sleep stages, as reported above. The time spent in each sleep stage (deep sleep, light sleep, and REM sleep) was expressed as a percentage of TST (%TST) of every participant to ensure that our data regarding deep sleep were comparable across all subjects. All individuals included in this analysis received detailed written and illustrated instructions on the proper use of the WatchPAT device. Subjects were instructed to activate the WatchPAT recorder just before bedtime and to deactivate it upon waking up.

Fifty-eight participants (64.0%) completed the WatchPAT evaluation during the first evaluation in the ALBION Cohort, coinciding with CSF collection and analysis. Additionally, 11 participants (12.0%) completed the WatchPAT evaluation within 1 year after the CSF evaluation, 13 participants (14.0%) within 2 years, 5 participants (5.5%) within 3 years, and 3 participants (3.3%) within 4 years.

### 2.5 Blood sampling and gene-apolipoprotein E (APOE) genetic outcomes

APOE genotyping was conducted using genomic DNA extracted from blood buffy coat, employing Qiamp DNA Blood Mini Kits (Qiagen, Venlo, Netherlands). The genotyping method utilized is polymerase chain reaction-DNA sequencing, carried out with LightCycler 2 (Roche Diagnostics GmbH) and LightMix TIB MOLBIOL reagents. Data from APOE genotyping analysis were available for 77 individuals. Participants with one or more APOE-ε4 alleles were considered APOE-ε4 carriers whereas those without APOE-ε4 alleles were regarded as APOE-ε4 non-carriers.

### 2.6 Statistical analysis

All analyses were performed with SPSS version 28.0. Statistical significance was set at *p* ≤ 0.050.

Continuous variables were expressed as mean ± SD or median with interquartile range (IQR), while categorical variables were represented as frequencies and percentages. Normality of all continuous variables was assessed using the Sharipo–Wilk normality test.

A positive family history of dementia was considered if either parent had late-onset dementia (after age 65); otherwise, it was considered negative.

The initial sample was stratified into two distinct sets of groups: first into MCI versus CN and subsequently into A+ versus A− groups. Differences in variables between CN subjects and MCI patients, as well as between amyloid negative (A−) and amyloid positive (A+) groups, were tested using independent samples *t*-test for continuous variables following the normal distribution (age, BMI, TST, CSF Aβ42 levels, percentage of deep sleep, percentage of light sleep, and percentage of REM sleep); otherwise, they were compared using independent samples – Mann Whitney *U-*test (education, ACE-R, MMSE, sleep latency, and number of awakenings). Chi-square (*X*²) test was used for comparing categorical variables (sex, APOE-ε4 carrier, family history).

Subsequently, two separate binary logistic regression analyses were conducted. One aimed to examine the association between deep sleep and cognitive function, while the other explored the relationship between deep sleep and amyloid-beta status. Regarding cognitive status, clinical diagnosis was selected as the outcome variable (CN = 0 and MCI = 1) with percentage of deep sleep, age, sex, and education years as predictor variables. Similarly, regarding amyloid-beta status, amyloid category was selected as the dependent variable (A− = 0/A+ = 1), with percentage of deep sleep, age, sex, and education years as independent variables. All independent variables were introduced in the model as continuous variables except for sex, which was treated as categorical variable (female as reference). Results are represented as odds ratio (OR) and 95% confidence interval (CI) of logistic regression coefficients.

In the unadjusted model, each independent variable was individually introduced into the regression analysis. In the adjusted model, all independent variables were simultaneously introduced into the regression analysis. The Omnibus tests of model coefficients were used to evaluate the extent to which the model improved over the baseline model. Nagelkerke *R* square value was used as a method of calculating the explaining variation. The Hosmer–Lemeshow test was used as a goodness of fit test. All binary logistic regression assumptions were checked.

A sensitivity analysis assessed the impact of different MCI types (aMCI and naMCI) on our results. After excluding naMCI individuals, we compared subjects based on their demographic, clinical, and sleep characteristics concerning cognitive status. Subsequently, we performed a corresponding regression analysis, with cognitive status as an outcome (CN = 0 and aMCI = 1) and age, sex, and years of education as predictor variables.

## 3 Results

### 3.1 Demographics, clinical characteristics, and sleep measures

#### 3.1.1 Cognitive status classification

A total of 90 individuals were included in our analysis. Patients’ demographic, clinical characteristics, and sleep measures are reported in Table 1.

**Table 1:** Demographics, clinical characteristics, and sleep measures classified by cognitive status

	All	CN	MCI	
	(*n* = 90)	(*n* = 72)	(*n* = 18)	*p-*value
Age (years), mean ± SD	63.43 ± 9.16	62.49 ± 9.06	67.22 ± 8.80	**0.049**
Sex, female (%)	63 (70.0)	52 (72.2)	11 (61.0)	0.358
Education (years), mean ± SD (min–max)	13.72 ± 3.85 (6–22)	14.06 ± 3.71 (6–22)	12.39 ± 4.20 (6–19)	0.158
ACE-R, mean ± SD (min–max)	92.21 ± 6.39 (61–100)	94.24 ± 4.04 (81–100)	84.11 ± 7.63 (61–94)	**<0.001**
MMSE, mean ± SD (min–max)	28.50 ± 1.84 (21–30)	29.01 ± 1.13 (25–30)	26.44 ± 2.59 (21–30)	**<0.001**
APOE-ε4 carrier, positive (%)	19 (24.6) [*n* = **77]**	13 (21.3) [*n* = **61]**	6 (37.5) [*n* = **16]**	0.181
Family history of dementia, positive (%)	49 (54.4)	41 (56.9)	8 (44.4)	0.341
CSF Aβ42 (pg/mL), Mean ± SD	1.221 (501) [*n* = 80]	1.276 (477) [*n* = 65]	983 (550) [*n* = 15]	0.040
BMI (kg/m^2^), mean ± SD	25.99 ± 3.96	25.69 ± 4.17	27.19 ± 2.79	0.154
TST (min), mean ± SD (min–max)	364 ± 74 (196–519)	356 ± 71 (196–498)	398 ± 79 (217–519)	**0.030**
Sleep latency (min), median (IQR)	19.00 (11.50)	19.00 (15.75)	20.00 (19.00)	0.481
Number of awakenings, median (IQR)	7.5 (5.25)	7 (5.00)	10 (12.50)	0.152
Percentage of deep sleep (%TST), mean ± SD	14.31 ± 5.69	15.18 ± 5.51	10.87 ± 5.16	**0.003**
Percentage of light sleep (%TST), mean ± SD	65.17 ± 12.16	63.73 ± 12.28	70.90 ± 10.06	**0.024**
Percentage of REM sleep (%TST), Mean ± SD	20.52 ± 8.69	21.09 ± 8.92	18.23 ± 7.53	0.214

All *p*-values are significant at the 0.050 level. Significant results are indicated with bold values.

CN, cognitively normal; MCI, mild cognitive impairment; ACE-R, Addenbrooke’s cognitive examination – revised; MMSE, mini-mental state examination; APOE, gene-apolipoprotein E; CSF, cerebrospinal fluid; BMI, body mass index; TST, total sleep time; REM, rapid eye movement.

Regarding cognitive status, 18 individuals were diagnosed with MCI, while 72 participants were classified as CN. No significant difference in sex ratio was found between the two groups. The participants with MCI were significantly older (*p* = 0.049) and, as expected, cognitive scores (MMSE, ACE) were lower in the MCI group compared to CN subjects (*p* < 0.001). Between the two groups, CSF Aβ levels were, on average, significantly lower in individuals diagnosed with MCI (*p* = 0.040). Regarding sleep characteristics, patients with MCI showed, on average, significantly longer TST by 42 min (*p* = 0.030), a lower percentage of deep sleep by 4.31% (*p* = 0.004), and a higher percentage of light sleep by 7.17% (*p* = 0.025) compared to CN individuals.

A sensitivity analysis, where only aMCI patients were included, showed that differences between the compared groups (CN/aMCI) regarding demographic, clinical characteristics, and sleep measures did not considerably alter and the statistically significant results remained unchanged (Table S1).

#### 3.1.2 Amyloid status classification

In total, 80 individuals successfully completed lumbar puncture and had available CSF biomarkers. Patients’ demographic, clinical characteristics, and sleep measures based on amyloid status are reported in Table 2.

**Table 2:** Demographics, clinical characteristics, and sleep measures classified by amyloid status

	All	Amyloid negative (A−)	Amyloid positive (A+)	
	(*n* = 80)	(*n* = 49)	(*n* = 31)	*p-*value
Age (years), mean ± SD	63.60 ± 8.95	61.41 ± 8.00	67.06 ± 9.39	***0.005***
Sex, female (%)	57 (71.2)	35 (71.4)	22 (70.9)	*0.965*
Education (years), mean ± SD (min–max)	13.64 ± 4.02 (6–22)	14.33 ± 3.98 (6–22)	12.55 ± 3.91 (6–22)	***0.027***
ACE, mean ± SD (min–max)	92.34 ± 6.61 (61–100)	93.39 ± 5.22 (80–100)	90.68 ± 8.17 (61–98)	*0.135*
MMSE, mean ± SD (min–max)	28.49 ± 1.88 (21–30)	28.57 ± 1.81 (22–30)	28.35 ± 2.01 (21–30)	*0.548*
APOE-ε4 carrier, positive (%)	17 (22.9) [***n*** **= 74]**	9 (19.5) [***n*** **= 46]**	8 (28.5) [***n*** **= 28]**	0.372
Family history of dementia, positive (%)	43 (53.7)	27 (55.1)	16 (51.6)	*0.760*
BMI (kg/m^2^), mean ± SD	25.73 ± 3.90	25.85 ± 4.08	25.55 ± 3.66	*0.742*
TST (min), mean ± SD (min–max)	364 ± 73 (196–504)	360 ± 72 (196–498)	369 ± 76 (217–504)	*0.595*
Sleep latency (min), median (IQR)	19.00 (7.00)	19.00 (16.00)	21.00 (11.00)	*0.059*
Number of awakenings, median (IQR)	7.5 (5.00)	7 (6.50)	8 (5.00)	*0.898*
Percentage of deep sleep (%TST), mean ± SD	14.45 ± 5.77	15.64 ± 5.70	12.56 ± 5.44	***0.019***
Percentage of light sleep (%TST), mean ± SD	65.41 ± 12.34	63.60 ± 12.63	68.26 ± 11.48	*0.100*
Percentage of REM sleep (%TST), mean ± SD	20.15 ± 8.77	20.76 ± 9.09	19.18 ± 8.30	*0.437*

All *p*-values are significant at the 0.050 level. Significant results are indicated with bold values.

ACE-R, Addenbrooke’s cognitive examination – revised; MMSE, mini-mental state examination; APOE, gene-apolipoprotein E; BMI, body mass index, TST, total sleep time, REM, rapid eye movement.

Thirty-one participants were classified as amyloid positive (A+). The amyloid positive group was, on average, significantly older by 5.65 years (*p* = 0.005) and had less years of education (*p* = 0.039) than the amyloid negative group (A−). Concerning sleep parameters, only the percentage of deep sleep was significantly different between groups. A+ subjects had on average, a significantly lower percentage of deep sleep by 3.08% (*p* = 0.019) than A− individuals.

### 3.2 Regression analysis

#### 3.2.1 Cognitive status

Results from multiple logistic regression are shown in Table 3. The unadjusted model revealed that for every 1% decrease in the percentage of deep sleep, there was approximately a 14% increase in the odds of being classified as MCI (95% CI [0.76, 0.96], *p* = 0.006). This association remained significant after controlling for age, sex, and years of education in the adjusted model (OR = 0.86, 95% CI [0.76–0.97], *p* = 0.012).

**Table 3:** Binary logistic regression analysis – cognitive status

Dependent variable: CN = 0, MCI = 1 CN (*n* = 72)/MCI (*n* = 18)						
	Unadjusted model			Adjusted model* adjusted for age, sex, and years of education		
Independent variables	OR	95% CI	*p*-value	OR	95% CI	*p*-value
Percentage of deep sleep (% TST)	0.86	0.76–0.96	**0.006**	0.86	0.76–0.97	**0.012**

*Adjusted model: Omnibus tests, *X*²(4) = 13.325, *p* = 0.01, Hosmer–Lemeshow test, *X*²(8) = 13.769, *p* = 0.09, Nagelkerke *R*² = 0.218.

All *p*-values are significant at the 0.050 level. Significant results are indicated with bold values.

Overall, after adjusting for potential confounders, a lower percentage of deep sleep was linked to an increased likelihood of MCI.

In sensitivity analysis excluding naMCI patients, logistic regression outcomes were similar (OR = 0.82, 95% CI [0.71–0.96], *p* = 0.011) in the adjusted model (Table S2).

#### 3.2.2 Amyloid status

As presented in Table 4, the unadjusted model showed that each 1% decrease in the percentage of deep sleep was associated, on average, with a 10% increase in the odds of being classified as A+ (OR = 0.90, 95% CI [0.83–0.99], *p* = 0.023). However, after controlling for age, sex, and years of education, the association became non-significant (OR = 0.92, 95% CI [0.84–1,01], *p* = 0.092).

**Table 4:** Binary logistic regression analysis – amyloid status

Dependent variable: amyloid negative = 0, amyloid positive = 1 A− (*n* = 49)/A*+* (*n* = 31)						
	Unadjusted model			Adjusted model* adjusted for age, sex, and years of education		
Independent variables	OR	95% CI	*p*-value	OR	95% CI	*p*-value
Percentage of deep sleep (% TST)	0.90	0.83–0.99	**0.023**	0.92	0.84–1.01	0.092

*Adjusted Model : Omnibus Tests, *X* ^2^(4) = 12.441 (*p* = 0.014) , Hosmer-Lemeshow test, *X* ^2^(8) = 10.077 (*p* = 0.26), Nagelkerke *R* ^2^ = 0.195.

All *p*-values are significant at the level of 0.050. Significant results are indicated with bold values.

Overall, after adjusting for potential confounders, a lower percentage of deep sleep was linked to a higher likelihood of amyloid positivity (A+); however, this association was not statistically significant at the 0.05 level.

## 4 Discussion

Overall, our analyses indicate that in non-demented individuals, a shorter duration of deep sleep, as a percentage of TST, is associated with higher odds of being MCI, even after controlling for age, sex, and years of education. In contrast, no statistically significant association between deep sleep and amyloid-beta status was found after adjusting for the above-mentioned confounding factors. Regarding other sleep measurements, in this study, subjects with MCI had a significantly longer TST compared to CN individuals. Additionally, the percentage of light sleep, defined as a combination of N1 and N2 NREM sleep stages, differed significantly among MCI and CN individuals; however, we considered this as a mirroring effect of the reduction of deep sleep stage and thus we did not further examine this parameter.

Our results regarding the relationship between MCI and deep sleep are consistent with several studies, although literature has also presented contradictory findings. Compared to this study, existing literature has primarily provided evidence from PSG studies involving participants predominantly over the age of 70, which contrasts with the younger age of participants in this study. First of all, Westerberg et al. [17] observed that individuals with aMCI spent less time in SWS compared to normal controls, suggesting a potential link between memory processing and deep sleep. However, the sample size of this analysis was considerably small, posing considerable limitations. Another study [18], which included CN individuals, aMCI, and AD subjects, revealed a significant difference in SWS percentage but *post-hoc* analysis indicated significance only in the comparison between CN and AD subjects, possibly due to the limited number of participants. Reda et al. [19] found a reduction in SWS percentage among those with aMCI compared to normal subjects and stated that this reduction in SWS could differentiate between cognitively impaired and CN older individuals. In a large cohort, with a high predominance of aMCI patients, Brunetti et al. [20] found that patients with MCI due to AD had a lower percentage of SWS compared to CN individuals. It is important to highlight the fact that in the aforementioned studies which found an association between MCI and deep sleep, the majority of the participants were classified as aMCI [17,18,19,20]. The majority of participants in this study were aMCI (66%) as well, which may explain why our findings are in accordance with similar studies. Specifically, our results remained robust after conducting a sensitivity analysis based on patients with aMCI despite the considerable limitations posed by the small number of aMCI cases, albeit predominant in this cohort (Supplementary material). Recently, a prospective study has associated reductions in SWS, as measured by PSG, with an increased incidence of dementia over a 17-year follow-up [44]. Similarly, a retrospective cohort study linked a decrease in deep sleep percentage to a higher risk of neurodegenerative disorders, such as AD [45]. Although the longitudinal nature of these studies provides evidence suggestive of causality, the absence of biomarkers, such as amyloid-beta42, which were included in our analysis, limits their ability to clarify the potential connection between deep sleep reduction and the underlying pathology of the disease.

In contrast, other studies [21,22,23,26] found no difference in SWS between individuals with different cognitive status. Kim et al. [21], although they did not report any differences between the MCI and CN regarding deep sleep percentage, noted that the amount of SWS was positively associated with the performance on language function tests in both groups. In comparison to our findings, another study [22] primarily reported differences in REM sleep, which were also associated with the APOE status of the individual. To a lesser extent they reported higher fragmentation of SWS in patients with MCI. Potential differences between our findings and these studies could be explained by the fact that we used a WatchPAT device to assess sleep stages instead of PSG, which provides a more thorough examination of sleep structure and architecture. Additionally, although these studies had larger sample sizes, they did not specify the MCI subtypes, such as aMCI and naMCI. Brayet et al. [24], in a study including patients with aMCI, concluded that the percentage of SWS did not significantly differ compared to CN individuals. Liguori et al. [26], in a large cohort, where CSF biomarkers were included, compared CN individuals, subjects with subjective cognitive impairment (SCI), patients with MCI due to AD, and patients with dementia of AD type. Despite observing a significant difference in the percentage of N3 (NREM sleep stage) across groups, a *post-hoc* analysis revealed no noticeable difference between MCI due to AD and CN individuals. In contrast to our study, this study provided evidence that REM sleep is altered in the preclinical stage of AD, namely MCI and subjects with SCI, and that it is linked to β-amyloid pathology and memory loss. Two meta-analyses were conducted on this topic as well; one regarding sleep measurements in all MCI groups and the other focusing on sleep structure exclusively in aMCI. A meta-analysis by D’Rozario et al. [27], which included several studies mentioned earlier, failed to identify differences in deep sleep characteristics between MCI and CN individuals. Another meta-analysis by Cai et al. [28], which included polysomnographic data on SWS from four studies, similarly found no difference between aMCI and CN, but it hinted at a trend toward lower deep sleep percentages in aMCI, while acknowledging the limitation of study’s sample size. However, apart from deep sleep percentage, authors reported a significant reduction in the total minutes of SWS between aMCI and CN individuals. However, in this study, we examined only the percentages of deep sleep, as we believe this approach is more consistent, reduces individual differences regarding TST, and enhances methodological accuracy. It is worth mentioning that these meta-analyses did not include newer studies on the topic [20,26].

The exact pathophysiological mechanism potentially connecting cognitive impairment to disrupted deep sleep remains unknown. The role of slow waves during deep sleep in the consolidation of memories has been investigated in cognitive normal individuals [13,14,16,46] noting the effects of SWS enhancement on various cognitive domains through auditory, electrical, or pharmaceutical stimulation in normal aging subjects. A potential link between the reduction of deep sleep and subsequent cognitive decline could involve amyloid beta (Aβ42). Sanchez-Espinosa et al. [29] found that an increased arousals index in SWS is associated with higher plasma Aβ42 in aMCI patients. However, AD pathophysiology is associated with lower (not higher) levels of Aβ42 in plasma and CSF [32] which makes the interpretation of this study as well as the comparison with our findings challenging. Additionally, it is important to highlight here that in contrast to the CSF amyloid biomarker included in our analysis, plasma Aβ42 may not be the most reliable plasma biomarker for AD pathology [47]. Another study by Varga et al. [30] revealed that in CN elderly, higher CSF Aβ42 values were associated with lower duration of SWS, in patients with normal CSF measurements. The authors proposed that disrupted deep sleep could potentially worsen the elevation of soluble brain Aβ levels prior to amyloid deposition. Meanwhile, Ju et al. [9] proposed that a lower percentage of deep sleep may result in increased amyloid in the interstitial space, thereby enhancing amyloid deposition and potentially leading to cognitive decline. According to this study, such a process could be explained by the fact that neurons tend to remain mostly silent, hyperpolarized, and exhibit reduced activity during deep sleep, whereas more amyloid beta is produced during periods of activation. Nevertheless, our results regarding amyloid status contradict this hypothesis, as a lower percentage of deep sleep did not significantly increase the odds of being A+ when confounders were adjusted for. This can be explained by the fact that amyloid-positive (A+) group was on average older than amyloid-negative (A−) group and it is generally accepted that age is associated with both reduction of time spent in deep sleep [8] and Aβ42 burden [48].

This study is not without limitations. First, due to the cross-sectional nature of this study, causality regarding the above-mentioned associations could not be determined. Second, there could be a selection bias as some of the participants were self-referred to the outpatient clinic, due to concerns about their memory. Furthermore, the gold standard technique used to acquire sleep data and accurately monitor sleep activity is polysomnography, which was not performed in this study. The sole validation study on sleep staging for WatchPAT [42] provided evidence for a specific age group younger than the cohort analyzed in this study. Therefore, the possibility of underestimating the deep sleep percentage in older adults due to inherent limitations of WatchPAT cannot be excluded. However, polysomnography is difficult to implement in population studies. Additionally, only 64% of our participants in this study completed WatchPAT sleep evaluation simultaneously (within a week) with lumbar puncture. This percentage increased to 90% when considering individuals that completed a WatchPAT study up to 3 years after CSF analysis. This mismatch in synchrony might have affected our implications, as Aβ42 values in CSF may not be constant over time. Finally, the relatively small sample size of this study, the notable predominance of CN individuals compared to MCI patients, 80 and 20%, respectively, and the fact that not all subjects selected were drug-naive regarding CNS-affecting medication might constrain the significance of our results.

Nevertheless, our approach possesses several strengths. First of all, our sample consisted of individuals who were, on average, younger (less than 70 years old) than participants in previous studies, allowing us to expand current knowledge regarding deep sleep during a crucial age zone concerning cognitive decline and Aβ42 aggregation. Second, we have adjusted our results for age considering the extensive literature regarding changes in sleep architecture, especially sleep stages, through normal aging [8]. Moreover, we presented both clinical and biomarker data from the same cohort, including a multifaceted sample of non-demented individuals and we used automated methods for CSF biomarkers assessment, which have demonstrated high agreement with amyloid PET scan imaging [49]. Additionally, WatchPAT is a portable device and thus a comfortable and affordable objective method for measuring sleep architecture, although not gold-standard. Clinical evaluation and neuropsychological assessment were conducted by experienced clinicians with subspecialty training and highly qualified neuropsychologists.

## 5 Conclusion

A potential bidirectional relationship between sleep and dementia is widely acknowledged in existing literature. However, the exact characteristics, the pathophysiology, and the prospective significance of such an association are yet to be fully determined. Deep sleep undoubtedly constitutes a fertile research field, given the emerging evidence on its role in memory consolidation, cognitive functions, and its interaction with amyloid beta (Aβ42). In this study, we explored the interplay between different aspects of AD continuum and deep sleep macroarchitecture in non-demented individuals. We found a considerable association between deep sleep and MCI. To confirm this evidence, longitudinal studies on non-demented patients should be conducted in order to unveil the relationship among disturbance in deep sleep patterns, cognitive decline, and biomarkers changes. After a comprehensive conjunction of these three fields, deep sleep could potentially serve as a new biomarker of cognitive decline as well as an open field for intervention for dementia prevention.

## Supplementary Material

Supplementary Table
