# Supplementary material for: Sleep architecture in Alzheimer’s disease continuum: The deep sleep question
Source: Open Life Sci. 2025 Mar 25;20(1):20251077. doi: 10.1515/biol-2025-1077 (PMC11947663; doi:10.1515/biol-2025-1077)
Supplement: Supplementary Table [file biol-2025-1077-sm.pdf]

Supplementary material

S1 MCI types – sensitivity analysis

In this analysis, we excluded non-amnestic MCI patients, namely six participants. Our sample constituted 72 cognitively normal individuals and 12 patients with amnestic mild cognitive impairment (aMCI). Tables S1 and S2 illustrate that the differences between CN individuals and those with aMCI did not significantly differ compared to disparities between CN and MCI subjects overall. Additionally, the results of the corresponding logistic regression remained robust compared to those from our main analysis. However, it is essential to note that the small number of aMCI participants in this analysis represents a notable limitation.

Table S1: Cognitive status classification; demographics, clinical characteristics and sleep measures

	**ALL** (***n*** **= 84)**	**CN** (***n*** **= 72)**	**aMCI** (***n*** **= 12)**	***p*-value**
Age (years), Mean ± SD	63.23 ± 9.08	62.49 ± 9.06	67.66 ± 8.19	0.067
Sex, female(%)	58 (69.0)	52 (72.2)	6(50.0)	0.123
Education (years), mean ± SD, (min–max)	13.92 ± 3.71 (6 – 22)	14.06 ± 3.71 (6–22)	13.08 ± 3.75 (6–18)	0.505
ACE-R, Mean ± SD, (min–max)	92.80 ± 6.13 (61–100)	94.24 ± 4.04 (81–100)	84.17 ± 9.09 (61–94)	<**0.001**
MMSE, Mean ± SD, (min–max)	28.69 ± 1.60 (22–30)	29.01 ± 1.13 (25–30)	26.75 ± 2.49 (22–30)	<**0.001**
APOE- ε4 carrier, positive (%)	18 (25.0) **[*n* = 72]**	13 (21.3) **[*n* = 61]**	5 (45.4) **[*n* = 11]**	0.089
Family history of dementia, positive(%)	47 (55.9)	41 (56.9)	6 (50.0)	0.654
BMI (kg/m^2), mean ± SD	26.04 ± 4.04	25.69 ± 4.17	28.10 ± 2.39	0.056
Total sleep time (min), mean ± SD (min–max)	364 ± 73 (196–518)	356 ± 71 (196–498)	415 ± 67 (296–518)	**0.009**
Sleep latency (min.), median (IQR)	19.00 (13.50)	19.00 (15.75)	20.00 (8)	0.826
Number of awakenings. median (Iqr)	7.5 (5.00)	7 (5.00)	10 (9)	0.052
Percentage of deep sleep(%TST), Mean ± SD	14.42 ± 5.74	15.18 ± 5.51	9.90 ± 5.14	**0.003**
Percentage of light sleep(%TST), Mean ± SD	64.90 ± 12.13	63.73 ± 12.28	71.91 ± 8.65	**0.030**
Percentage of REM Sleep(%TST), Mean ± SD	20.68 ± 8.63	21.09 ± 8.92	18.19 ± 6.39	0.283

All *p*-values are significant at the 0.050 level. Significant results are indicated with bold values.

CN cognitively normal, aMCI amnestic mild cognitive impairment, ACE-R addenbrooke's cognitive examination - revised , MMSE mini-mental state examination, APOE gene-apolipoprotein E , BMI body mass index , TST total sleep time , REM rapid eye movement.

Table S2: Binary logistic regression analysis – cognitive status

Dependent variable: cognitively normal = 0 , amnestic mild cognitive impairment = 1 *CN (n= 72) / aMCI (n= 12)*						
	**Unadjusted model**			**Adjusted model* adjusted for age, sex, years of education**		
**Independent variables**	**OR**	**95% CI**	***p*-value**	**OR**	**95% CI**	***p*-value**
Percentage of deep sleep, (% TST)	0,82	0,72–0,94	**0.005**	0,82	0,71–0,96	**0.011**

*Adjusted model : Omnibus Tests, $X^2(4) = 13.308$, $p = 0.01$, Hosmer-Lemeshow test, $X^2(8) = 7.054$, $p = 0.53$, Nagelkerke $R^2 = 0.262$.

All *p*-values are significant at the 0.050 level. Significant results are indicated with bold values.
